# Editorial: Current Concepts of Cellular and Biological Drugs to Modulate Regulatory T Cell Activity in the Clinic

**DOI:** 10.3389/fimmu.2016.00141

**Published:** 2016-04-14

**Authors:** Helmut Jonuleit, Tobias Bopp

**Affiliations:** ^1^Department of Dermatology, University Medical Center Mainz, Johannes Gutenberg-University, Mainz, Germany; ^2^Institute of Immunology, University Medical Center Mainz, Johannes Gutenberg-University, Mainz, Germany

**Keywords:** regulatory T cells, dendritic cells, immunoregulation, autoimmunity, autoimmune diseases, clinical studies

**The Editorial on the Research Topic**

**Current Concepts of Cellular and Biological Drugs to Modulate Regulatory T Cell Activity in the Clinic**

Regulatory T (Treg) cells are essential for the maintenance of peripheral tolerance and prevent the development of autoimmunity and allergy. While on the one hand being indispensable for the perpetuation of tolerance to harmless antigens or self-antigens, Treg cells contribute to cancer pathogenesis and progression (1). Hence, the potential to treat a multitude of different human diseases by pharmacological modulation of Treg cells is enormous. Consequently, this T cell population is in the focus of biomedical research and development. Currently, isolated and *in vitro* expanded Treg cells as cellular therapeutics are intensively tested in clinical trials for their ability to prevent and ameliorate graft-versus-host disease (GvHD) and first biologicals to modulate Treg cell activity directly in man are being developed and tested in preclinical and clinical trials.

This Research Topic comprised seven review articles; the authors discuss selected developments on the way from basic research on manipulation of peripheral tolerance mechanisms toward clinical application.

Mahnke et al. present an updated view on the critical role of dendritic cells (DC) in control and maintenance of peripheral tolerance under non-inflammatory conditions based on findings obtained in preclinical mouse models showing that factors of local microenvironment and extrinsic inflammatory signals dictate the function of these “sentinels of immunity” (2): inducers of tolerance versus immunity. Analogous to this overview of basic DC research in the mouse, Raker et al. provide an overview of translational research on human tolerogenic DC, the important role of IL-10 for stability of their phenotype and their potential as cellular therapeutics for reestablishment of peripheral tolerance in patients with autoimmune or allergic diseases (Raker et al.). Hahn et al. discuss current developments in establishment of humanized mouse models for preclinical testing of novel biologicals to manipulate Treg cell suppressive function *in vivo*. Additionally, the risk and benefit of cellular therapies using *in vitro* expanded Treg cells or pharmacological modulators of Treg cell function like rapamycin or IL-2 is comparatively discussed by Perdigoto et al. Complementary, Pham et al. provide an updated view on the use of low dose IL-2 in combination with tissue-specific antigens as a therapeutic approach to expand and activate antigen-specific Treg cells in patients with type I diabetes (Pham et al.), an approach that will be currently used by different groups for treatment of T cell-mediated autoimmunity. A well-known strategy for tolerance induction first described by Hermann Waldman’s group in 1990 is the application of non-depleting anti-CD4 antibodies that induces a long lasting antigen-specific tolerance in mouse models of solid organ transplantation (3). In this Research Topic, König et al. report on their clinical experiences by using a humanized anti-CD4 antibody (BT-061, Tregalizumab) for treatment of rheumatoid arthritis in patients. This anti-CD4 mAb, first tested in patients with psoriasis, induces suppressive function of human Treg without blocking T helper cell activities *per se*. Additionally, Hui et al. discuss recent advances of management of advanced non-small cell lung cancer by PD-1-blocking antibodies and their impact on cytokine-induced killer cell activity. In summary, this Research Topic of Frontiers in Immunology provides a snapshot of our recent understanding of Treg cell biology and function with a special focus on current concepts of therapeutic strategies to modulate peripheral tolerance mechanisms and Treg cell activity in humans. Given the recent advances in our understanding of the molecular mechanisms underlying cell type- and ­tissue-specific Treg cell function, it seems promising that Treg cell-based cellular therapeutics as well as novel biologicals to directly and cell-specifically modulate Treg cell function are entering the clinic, and first results of their potential as tolerance modifiers will be available in near future.

## Author Contributions

All authors listed, have made substantial, direct and intellectual contribution to the work, and approved it for publication.

## Conflict of Interest Statement

The authors declare that the research was ­conducted in the absence of any commercial or financial relationships that could be construed as a potential conflict of interest.
